# Laxative use in adults with intellectual disabilities: development of prescribing guidelines

**DOI:** 10.1192/bjo.2024.50

**Published:** 2024-04-18

**Authors:** Ruth Bishop, Richard Laugharne, Lisa Burrows, CHAMPS Team, Sandra Ward, Sharon Eustice, David Branford, Mike Wilcock, Kirsten Lamb, Alison Tavare, Charlotte Annesley, Stephen Lewis, Vasileios Voulgaropoulos, Faye Sleeman, Beth Sargent, Rohit Shankar

**Affiliations:** Cornwall Intellectual Disability Equitable Research (CIDER), Cornwall Partnership NHS Foundation Trust, Truro, UK; and CIDER, University of Plymouth Peninsula School of Medicine, Truro, UK; CIDER, Cornwall Partnership NHS Foundation Trust, Truro, UK; and CIDER, University of Plymouth Peninsula School of Medicine, Truro, UK; Knowledge Spa Library, Royal Cornwall Hospital Trust, Truro, UK; and The Southwest Clinical School, University of Plymouth, Truro, UK; The Cornwall Intellectual Disability and Autism Support Team, Cornwall Council, Truro, UK; Independent expert with lived experience, Truro, UK; CIDER, Cornwall Partnership NHS Foundation Trust, Truro, UK; CIDER, University of Plymouth Peninsula School of Medicine, Truro, UK; Royal Cornwall Hospital Trust, Truro, UK; Health Innovation West of England, Bristol, UK; Learning Disability Liaison Service, North Middlesex University Hospital NHS Trust, London, UK; Gastroenterology Department, University Hospitals Plymouth NHS Trust, Plymouth, UK; Pharmacy Department, Hertfordshire Partnership University NHS Foundation Trust, Hatfield, UK; Bowden Derra Park, Launceston, UK

**Keywords:** Carers, comorbidity, education and training, intellectual disability, patients

## Abstract

**Background:**

Constipation is overrepresented in people with intellectual disabilities. Around 40% of people with intellectual disabilities who died prematurely were prescribed laxatives. A quarter of people with intellectual disabilities are said to be on laxatives. There are concerns that prescribing is not always effective and appropriate. There are currently no prescribing guidelines specific to this population.

**Aims:**

To develop guidelines to support clinicians with their decision-making when prescribing laxatives to people with intellectual disabilities.

**Method:**

A modified Delphi methodology, the RAND/UCLA Appropriateness Method, was used. Step 1 comprised development of a bespoke six-item, open-ended questionnaire from background literature and its external validation. Relevant stakeholders, including a range of clinical experts and experts by experience covering the full range of intellectual disability and constipation, were invited to participate in an expert panel. Panel members completed the questionnaire. Responses were divided into ‘negative consensus’ and ‘positive consensus’. Members were then invited to two panel meetings, 2 weeks apart, held virtually over Microsoft Teams, to build consensus. The expert-by-experience group were included in a separate face-to-face meeting.

**Results:**

A total of 20 people (ten professional experts and ten experts by experience, of whom seven had intellectual disability) took part. There were five main areas of discussion to reach a consensus i.e. importance of diagnosis, the role of prescribing, practicalities of medication administration, importance of reviewing and monitoring, and communication.

**Conclusions:**

Laxative prescribing guidelines were developed by synthesising the knowledge of an expert panel including people with intellectual disabilities with the existing evidence base, to improve patient care.

Constipation is a common disorder that involves difficulties with defecation. This may be experienced as infrequent and/or hard stools, difficulty passing stools, or the sensation of incomplete emptying or anorectal blockage.^1^ Constipation is a symptom-based disorder affected by diet, fluid intake, medication, exercise and toilet habits.^2^

## People with intellectual disabilites and constipation

People with intellectual disabilities are more likely to suffer from constipation than people without an intellectual disability.^3^ The antecedents and aetiology are multifactorial, as increased risk may be attributable to medication, suboptimal diet, low physical activity levels or poor mobility.^4,6^ The issue of constipation for people with intellectual disabilities is a significant problem that causes suffering and can even lead to death.^7,8^

The main management response in this client group is with laxatives.^4^ It has been reported that a quarter of people with an intellectual disability are on regular laxatives, compared with 0.5% of the general population.^9^ The guidance for laxative use in people with intellectual disabilities is currently the same as for the general population,^10^ and consists of the National Institute for Health and Care Excellence (NICE) prescribing information for laxatives.^11^ The NICE guidelines suggest lifestyle advice and review of secondary causes are considered before using laxatives.

## Limitations

Laxatives come in four main classes: bulk-forming, osmotic, stimulant and softeners.^12^ Bulk-forming laxatives increase the ‘bulk’ or weight of the stool, which stimulates the bowel.^12^ Osmotic laxatives draw water from the rest of the body into the bowel to soften the stool and make it easier to pass.^12^ Stimulants stimulate the muscles that line the gut, helping the stool to move along.^12^ Softeners let water into the stool to make it softer and easier to pass.^12^ The specific laxative classes and the problems they can cause people with intellectual disabilities is enumerated in Table 1.

**Table 1:** Classes of oral laxatives

Class	Examples	Issues for people with intellectual disabilities
Softener	Docusate	Often needs additional laxative from other class Side effects: cramps and diarrhoea
Stimulant	Bisacodyl (oral and suppository available), senna, sodium picosulphate	Need stool to be softened (fluids, fibre, softener) Avoid in obstruction Side effects: cramps and diarrhoea
Osmotic	Polyethylene glycols (macrogols – Movicol, Laxido, Cosmicol), lactulose	Need good fluid intake or can get dehydration Side effects: flatulence, bloating, cramps, nausea Patients with dysphagia: problem with macrogols combining with starch-based thickeners, but not xanthan gum-based thickeners. How to make up macrogol laxatives: https://www.youtube.com/watch?v=HCI84ifbGFI
Bulk forming	Ispaghula (Fybogel), psyllium	Can lead to blockage, needs good fluid intake to prevent obstruction
Others	Prucalopride	Only prescribed by gastroenterologists in secondary care, when other treatments have failed

There is a lack of high-quality evidence about how effective laxatives are, and whether certain laxatives are better than others.^13^ Alongside the NICE guidelines there are local resources that have been developed within the National Health Service (NHS) to support laxative prescribing in the general population.^14^

The scientific evidence relating to laxative use with people with intellectual disability is limited. The literature that is available suggests poor prescribing practices and recommends developing guidelines specifically for this population group.^8,15^ A wide range of laxatives are used in the intellectual disability population, and laxative polypharmacy is common.^4,6^ Laxatives appear often to be used inappropriately and given in an unstructured way;^4,8^ for example, prescribing two laxatives from the same class or being prescribed laxatives long term with no obvious effect for the patient.

There are no specific national guidelines for prescribing laxatives to adults with intellectual disabilities.^16^ Prescribing laxatives can be complex in this population group. As with many other areas relating to the health of people with intellectual disabilities, there is very limited research available and clinical practice is informed by research from the general population.^17,18^

To address the need to improve laxative prescribing practices, the aim of the project was to develop specific laxative prescribing guidelines for adults with intellectual disability.

## Method

### Study design and questionnaire development

A modified Delphi methodology, the RAND/UCLA Appropriateness Method (RAM), was selected to collect different opinions from specialists and experts with lived experience (patients and their carers) through multiple rounds to possibly determine consensus.^19^ The RAM is a formal group consensus process that systematically and quantitatively combines expert opinion and evidence by asking panellists to rate, discuss and then re-rate items.^19^ RAM is a modified Delphi method that provides panellists with the opportunity to discuss their judgements between the rating rounds. RAM has been used to develop clinical practice guidelines in a variety of areas.^20^

The formulation of a questionnaire represented the initial step, designed by the core team comprising the researcher (R.B.) and two clinical experts (R.L. and R.S.), supported by an information specialist (L.B.) who provided background literature.

### Literature review

A rapid review was adopted to provide a time-based overview of the current state of knowledge of laxative prescribing for people with intellectual disabilities. Biomedical database of PubMed (as prescribing was felt to be a medical issue) was searched with terms ‘intellectual disabilities AND laxatives AND guidelines’. The shortlisting inclusion criteria required that the paper discuss laxative use in people with intellectual disabilities. For a paper to be fully included it needed to provide information on laxative guidelines or best practice recommendations of its use in adults with intellectual disabilities. No time limits were placed. Searches were done only in English. Further additions were identified through a combination of web searches of grey literature and conversations with expert panel members. The core team engaged in a collaboration to critically evaluate the identified literature as well as their own experience in clinical practice, focusing on possible debate points of the subject matter. The objective was to formulate a suitable qualitative questionnaire with six open-ended questions that aimed to generate ideas and elicit opinions on laxative prescribing for adults with intellectual disabilities. The design of the questionnaire was based on the findings of a literature review and an existing, regionally produced guideline for the general population. Once developed, the questionnaire was evaluated by three external validators (MW – pharmacist, KL – general practitioner, SE – specialist incontinence nurse) chosen by the core team to test its understandability and clarity. The study was carried out between May and July 2023. The final questionnaire is presented in Supplementary File 1 available at https://doi.org/10.1192/bjo.2024.50.

### Expert panel

Expert panels combine scientific evidence and clinician expertise to provide evidence-based information to guide clinical decision-making.^21^ Relevant stakeholders covering the full range of intellectual disability and constipation were invited to participate in an expert panel to develop the prescribing guidelines. The panel members provided a range of experience in intellectual disabilities and/or laxative prescribing. Members were identified through professional networks. Panel members involved significant representation by people with intellectual disability, i.e. Cornwall Health and Making Partnerships (CHAMPS) team as peer researchers, care providers with lived experience (professional and family), specialists (psychiatrists/general practitioners/specialist physician) in managing biopsychosocial problems in people with intellectual disabilities, pharmacists, a gastroenterologist and a specialist continence nurse.

Our peer researchers (CHAMPS team) are people with lived experience. To engage them suitably in this project, the CHAMPS team were given training on the project goals and expectations, and what meaningful engagement from them would look like. They were provided renumeration for their time and have been recognised as significant contributors to the project by inclusion as co-authors.

Because of international differences in healthcare delivery, the panel included only experts who were based in the UK.

### Collating expert opinion and consensus determination

Panel members were initially invited to complete the questionnaire and responses were collected with the online software Microsoft Forms for Windows. The questionnaire included an embedded link to the NICE prescribing information for laxatives^11^ to orientate the panel members to the focus of the project. Responses were divided into two segments for the research purposes: ‘negative consensus’ and ‘positive consensus’.

Members were then invited to two panel meetings, 2 weeks apart, held virtually over Microsoft Teams, version 4.2.4.0 for Windows. The first of the two meetings involved a discussion on the questionnaire responses with a view to build consensus. The information generated was used to draft an initial outline of laxative prescribing guidelines for adults with intellectual disabilities. and adapted using the expertise of the panel. The second online meeting was used for comment and feedback on the draft guidelines.

An expert-by-experience group employed by the local authority were invited to take part in the expert panel. Members of the group have intellectual disabilities and/or autism. They were invited to complete the questionnaire and then attend the two online panel meetings. The group were unable to attend the online panel (because of the time and date) and so were included in a separate face-to-face meeting. Author R.B. met the expert-by-experience group and shared the draft guidelines developed. The group were given the opportunity to share expertise and the guidelines were adapted accordingly.

A final draft document based on the expert views, clinical experience and current evidence was developed. This was shared with the expert panel by email for review, and the draft was updated to reflect feedback gathered. Once consensus was reached the guidelines were finalised.

### Ethics and governance

This investigation excluded human patient involvement or patient data processing, and so ethical endorsement was not necessary. All panel members provided their informed consent to join the panel. Further, those people with intellectual disability taking part in the process were doing so as peer researchers and consented fully to do so.

## Results

### Literature review

The literature review in PubMed led to eventual shortlisting of 12 papers, and 11 other non-indexed linked articles were found from other searches and inquires. A full list of these is provided in Supplementary File 2. None of the 23 articles directly dealt with the core inquiry of best practice in laxative prescribing for adults with intellectual disabilities. Where mentioned, reference was made to use laxatives as for the general population. These articles, however, contributed to the project discussions.

### Expert panel

A total of 20 people, including ten professional experts and ten experts with lived experience (of whom seven had intellectual disabilities) took part in the panel. Table 2 shows a breakdown of who completed the questionnaire and attended the meetings. The outcome of the process, i.e. the proposed guidelines, is presented in Appendix 1. There were five main areas of panel discussion to reach a consensus: importance of diagnosis, the role of prescribing, practicalities of medication administration, importance of reviewing and monitoring, and communication.

**Table 2:** Participation of the expert panel

Role	Completed questionnaire	Attended session one (online panel session)	Attended session two (online panel session)	Attended face-to-face meeting
Neuropsychiatrist	No	Yes	Yes	No
Psychiatrist	Yes	Yes	Yes	No
General practitioner	Yes	Yes	No	No
Physician	No	Yes	Yes	No
Pharmacist	Yes	Yes	Yes	No
General practitioner	No	Yes	Yes	No
Care staff (two participants)	Yes	Yes	Yes	No
Experts by experience	Yes	No	No	Yes
Gastroenterologist	Yes	No	Yes	No
Pharmacist	Yes	Yes	No	No
Bladder and bowel nurse	Yes	No	No	No
Pharmacist	Yes	Yes	No	No
Parent carer	Yes	Yes	No	No
Total	10	10	7	7

### Correct diagnosis

Before prescribing laxatives it is necessary to ensure that a correct diagnosis of constipation is made. This requires a holistic assessment and understanding of potential causes of constipation relevant to this population, such as side-effects of medications. It is at this stage that red flags need to be identified and considered. It also needs to be acknowledged that not every client will understand what constipation is or understand medical terminology.

Once constipation is confirmed and potential causes identified, an individualised treatment plan can be developed. Non-pharmacological methods need to be implemented before laxative use, if possible.

### Prescribing

To support decision-making, it was suggested that a stepwise algorithm be developed with gradual dosing to avoid overprescribing. The algorithm has two routes, depending on the mobility of the patient.

### Practicalities of administration

The practicalities of taking laxatives were identified as an area of importance. This included considering how these medications taste or how they can be administered to those with swallowing difficulties. The important role of including carers in the monitoring of constipation, as well as administering laxatives, was emphasised.

### Reviewing and monitoring

It is important an individual's laxative use, including over-the-counter laxatives, and constipation are regularly reviewed. There are various ways monitoring could take place, including the annual health check, community pharmacists and by carers.

### Communication

When prescribing laxatives, good communication between the clinician and the client and/or carer is vital for effective laxative use. Whether laxatives are self-administered or administered with support of a carer, the treatment plan needs to be explained so that the individual or carer can support the monitoring and review of laxatives. Poor communication can lead to medications not being taken properly or a poor understanding of the reasons for taking the medication.

## Discussion

People with intellectual disabilities are predisposed to bowel problems because of higher levels of multimorbidity and polypharmacy.^22,25^ Constipation is a significant issue for the intellectual disability population, and can lead to serious complications.^16^ The England Learning Disability Mortality Review has highlighted that nearly a quarter of people with intellectual disabilities who were reviewed for premature mortality had constipation.^26^ A further report conducted focused reviews (*n* = 97) on their 2021 mortality data.^27^ It found that almost half of the people in the focus review had problems with constipation.^27^ Over half of the sample were prescribed medications that could cause constipation.^27^ The review found that 40% of their sample were prescribed laxatives.^27^ There was further evidence to suggest a lack of knowledge of suitable education and communication of normal bowel function and habits.^27^ In response to this significant finding, NHS England produced the constipation campaign toolkit to help raise awareness of the concerns.^28^ However, there has been little direct evidence to address the concerns on laxative prescribing.

The main management strategy of management of constipation is with laxatives, but the evidence available demonstrates that laxative prescribing in this population is not well managed.^8,27^ The current picture suggests there is a need for the laxative prescribing guidelines proposed in this paper for adults with intellectual disabilities, so that those prescribing laxatives can be enabled to make good clinical decisions. The guidelines were developed by synthesising the knowledge of the panel with the existing knowledge base. Recent studies have recognised that those with lived experience have found it frustrating getting their views heard with regards to people with intellectual disabilities and constipation.^29^ The inclusion of experts by experience in the form of those with intellectual disabilities, family members and formal carers in equal numbers to health professionals, have enabled the guidelines to be informed by a diverse range of experiences and expertise, which should be favourable for its adoption in practice. The produced guidelines give further detail and information about considerations needed when prescribing laxatives to adults with an intellectual disability, when compared with the existing NICE guidelines for the general population.^11^

### Limitations

This study included people with intellectual disability currently employed in the local authority, but it could be argued that this group is not representative of the intellectual disability population, as they have mild-to-moderate intellectual disability. This concern is mitigated in part by the inclusion of both family and formal carers in the study who have experience of supporting individuals with more severe intellectual disability presentations, allowing the perspective of a wide range of concerns to be considered during the guideline development.

Modifying the Delphi method could be seen as a limitation of the study. However, the use of a modified Delphi method was selected in part to enable inclusion of a wide range of experience and knowledge in formulation of the guidelines. By allowing for in-person meetings and online discussions, voices of those with lived experience were able to be combined with the voices of individuals with professional expertise and experience.

### Implications for clinical practice

The guidelines have been developed to be concise and straightforward to use for prescribing clinicians. Management of constipation in adults with intellectual disabilities needs to be individualised and based on an understanding of the factors influencing a person's constipated state. This is best recorded in an individualised bowel care plan, which is reviewed regularly.^6^

### Implications for research

There are ethical limitations on the appropriateness of certain research designs, as it would be difficult to justify comparing laxatives with placebo in this patient group, as the placebo group may be at risk of suffering. However, trials comparing the efficacy of different laxatives or combinations of laxatives may increase knowledge. There is also a need for research on the length of time laxatives need to be prescribed and on de-prescribing. The active use of individual bowel care plans needs to be evaluated to see if this improves patient outcomes.

### Implications for policy

These guidelines need to be considered regarding their suitability for rolling out as advice for prescribing in this client group. We are not aware of any other specific guidelines, and we hope this can be the first attempt at helping clinicians prescribe appropriately for this vulnerable population.

## Supporting information

Bishop et al. supplementary material 1 Bishop et al. supplementary material

Bishop et al. supplementary material 2 Bishop et al. supplementary material

## Data Availability

The data that support the findings of this study are available from the corresponding author, R.S., upon reasonable request.

## Appendix 1 Laxative use in adults with intellectual disability: prescribing guidelines

These general guidelines are written to help in prescribing laxatives, but best practice is for all patients to have an individualised bowel care plan.
