# Supplementary material for: Laxative use in adults with intellectual disabilities: development of prescribing guidelines
Source: BJPsych Open. 2024 Apr 18;10(3):e84. doi: 10.1192/bjo.2024.50 (PMC11060064; doi:10.1192/bjo.2024.50)
Supplement: Bishop et al. supplementary material 1 — Bishop et al. supplementary material [file S2056472424000504sup001.pdf]

Laxative Prescribing Guidelines for People with Intellectual Disabilities

People with intellectual disabilities are more likely to suffer from constipation than people without an intellectual disability. One of the treatment options for constipation are laxatives. We are seeking your thoughts on laxative use in people with intellectual disabilities so that prescribing guidelines for this population group can be developed.

This questionnaire forms round one of a three round process. The second and third rounds are the planned meetings on the 24th May and the 6th June. We aim to develop a consensus on what these guidelines will include by the end of the third round.

Please answer the questions below as fully as you can. The responses will be collated and used to shape the discussion in round two.

* Required

* This form will record your name, please fill your name.

1. Please enter your name and job title *

2. Currently the guidelines for laxative use in people with intellectual disabilities is the same as for the general population. We would first welcome your feedback on the existing NICE guidelines for laxatives (https://cks.nice.org.uk/topics/constipation/prescribing-information/) and their relevance to people with intellectual disabilities. Please use the space below to discuss your thoughts including details of any modifications, if any, you think are needed.

   *

3. What needs to be considered when someone with an intellectual disability uses a laxative? *

4. What difference, if any, will an individual's severity of intellectual disability have on laxative use? *

5. 'There's some evidence that for people with intellectual disabilities there can be an over reliance on laxatives'. What are your thoughts and experience of this? *

6. Who should monitor laxative use? How often should this happen? *

7. Please use the space below to add any other thoughts you have on this issue.

This content is neither created nor endorsed by Microsoft. The data you submit will be sent to the form owner.

Microsoft Forms
