# Supplementary material for: Laxative use in adults with intellectual disabilities: development of prescribing guidelines
Source: BJPsych Open. 2024 Apr 18;10(3):e84. doi: 10.1192/bjo.2024.50 (PMC11060064; doi:10.1192/bjo.2024.50)
Supplement: Bishop et al. supplementary material 2 — Bishop et al. supplementary material [file S2056472424000504sup002.docx]

**Literature review shortlisted papers**

1. **From indexed database search**
2. Maslen, C., Hodge, R., Tie, K., Laugharne, R., Lamb, K., & Shankar, R. (2022). Constipation in autistic people and people with learning disabilities. *The British journal of general practice : the journal of the Royal College of General Practitioners*, *72*(720), 348–351. <https://doi.org/10.3399/bjgp22X720077>
3. National Institute for Health and Care Excellence. (2023b). Constipation: what is it? Retrieved from: <https://cks.nice.org.uk/topics/constipation/background-information/definition/>
4. Emly, M., & Marriott, A. (2017). Revisiting constipation management in the community. British journal of community nursing, 22(4), 168–172.
5. Robertson, J., Baines, S., Emerson, E., & Hatton, C. (2018). Prevalence of constipation in people with intellectual disability: A systematic review. Journal of Intellectual & Developmental Disability, 43 (4), 392-406.
6. Robertson, J., Baines, S., Emerson, E., & Hatton, C. (2018). Constipation management in people with intellectual disability: A systematic review. *Journal of applied research in intellectual disabilities: JARID*, *31*(5), 709–724. <https://doi.org/10.1111/jar.12426>
7. Laugharne, R., Wilcock, M., Rees, J., Wainwright, D., Newton, N., Sterritt, J., Badger, S., Bishop, R., Bassett, P., & Shankar, R. (2023). Clinical characteristics of people with intellectual disability admitted to hospital with constipation: identifying possible specific high-risk factors. Journal of intellectual disability research : JIDR, 10.1111/jir.13108. Advance online publication. <https://doi.org/10.1111/jir.13108>
8. Laugharne R, Sawhney I, Perera B, Wainwright D, Bassett P, Caffrey B, O’Dwyer M, Lamb K, Wilcock M, Roy A, Oak K, Eustice S, Newton N, Sterritt J, Bishop R, Shankar R (2024) Chronic constipation in people with intellectual disabilities in the community: a cross-sectional study British Journal of Psychiatry Open (in press)
9. Roy A & Simon GB (1987). Intestinal obstruction as a cause of death in the mentally handicapped. Journal of Intellectual Disability Research, 31, 193-197.
10. AlMutairi, H., O’Dwyer, M., Burke, E., McCarron, M., McCallion,.P., & Henman, M.C. (2020). Laxative use among older adults with intellectual disability: a cross sectional observational study. International Journal of Clinical Pharmacy, 42, 89-99.
11. Carey, I.M., Hosking, F.J., Harris, T., DeWilde, S., Beighton, C., & Cook, D.G. (2017). An evaluation of the effectiveness of annual health checks and quality of health care for adults with intellectual disability: an observational study using a primary care database. Health Services Delivery Research, 5 (25).
12. Public Health England. (2016). Constipation: making reasonable adjustments. Retrieved from: <https://www.gov.uk/government/publications/constipation-and-people-with-learning-disabilities/constipation-making-reasonable-adjustments>
13. National Institute for Health and Care Excellence. (2023a). Constipation: Prescribing information. Retrieved from: <https://cks.nice.org.uk/topics/constipation/prescribing-information/>
14. **From other searches**
15. NHS. (2022). Laxatives. Retrieved from: <https://www.nhs.uk/conditions/laxatives/>
16. NHS Inform. (2023). Laxatives. Retrieved from: [https://www.nhsinform.scot/tests-and-treatments/medicines-and-medical-aids/types-of-medicine/laxatives#:~:text=Considerations-,Introduction,exercise%2C%20haven't%20helped](https://www.nhsinform.scot/tests-and-treatments/medicines-and-medical-aids/types-of-medicine/laxatives#:~:text=Considerations-,Introduction,exercise%252C%2520haven't%2520helped)
17. HERPC [Hull and East Riding Prescribing Committee] (2019) Management of Constipation in Adults. <https://www.hey.nhs.uk/wp/wp-content/uploads/2019/08/GUIDELINE-Constipation-guidelines-updated-may-19.pdf>
18. SCCG [Sunderland Clinical Commissioning Group] (2018) Primary Care Laxative Guidelines for Adults. <https://www.sunderlandccg.nhs.uk/wp-content/uploads/2018/03/SCCG-Laxative-Guideline-v2.0-2.pdf>
19. Summerfield, (2015) Rotherham Clinical Commissioning Group Laxative Guidelines for Adults. <http://www.shakespeareroadsurgery.co.uk/files/Medicines%20Management/Guidelines/Laxative%20Guidelines%20Nov%202013.pdf>
20. SCW. (2022). Review of constipation in people with a learning disability and autistic people: Summary Report. Retrieved from: <https://www.scwcsu.nhs.uk/documents/66-summary-report-review-of-constipation-in-people-with-a-learning-disability-and-autistic-people-v1-1/file>
21. LeDeR annual report 2019 <https://leder.nhs.uk/images/annual_reports/LeDeR_2019_annual_report_FINAL2.pdf> accessed (28/01/2024)
22. LeDeR annual report 2022 <https://www.kcl.ac.uk/ioppn/assets/fans-dept/leder-2022-v2.0.pdf> (accessed 28/01/2024)
23. NHS England Constipation campaign toolkit <https://www.england.nhs.uk/long-read/constipation-campaign-toolkit/> (accessed 28/01/2024)
24. Pouard T (2023) Constipation in people with learning disabilities: prevalence and impact. Nursing Times [online]; 119: 4.
25. <https://www.england.nhs.uk/publication/national-primary-care-clinical-pathway-for-constipation-in-children/>
